# Incidental Findings in Lung Cancer Screening

**DOI:** 10.3390/cancers16142600

**Published:** 2024-07-20

**Authors:** Yenpo Lin, Khulan Khurelsukh, I-Gung Li, Chen-Te Wu, Yi-Ming Wu, Gigin Lin, Cheng-Hong Toh, Yung-Liang Wan

**Affiliations:** 1 Department of Medical Imaging and Intervention, Chang Gung Memorial Hospital at Linkou, Taoyuan City 333, Taiwan; yenpojack@cgmh.org.tw (Y.L.); melik@cgmh.org.tw (C.-T.W.); m7075@cgmh.org.tw (Y.-M.W.); giginlin@cgmh.org.tw (G.L.); eldomtoh@cgmh.org.tw (C.-H.T.); 2 Department of Medical Imaging and Radiological Sciences, College of Medicine, Chang Gung University, Taoyuan City 333, Taiwan; h_ashka@yahoo.com; 3 Department of Medical Imaging and Intervention, New Taipei Municipal Tucheng Hospital, New Taipei City 236, Taiwan; ladenli@cgmh.org.tw

**Keywords:** lung cancer screening, low-dose computed tomography, incidental findings

## Abstract

**Simple Summary:**

Low-dose computed tomography (LDCT) scans often reveal incidental findings (IFs) not directly associated with lung cancer screening (LCS). These findings can pose challenges in LDCT interpretation and clinical management. This narrative review examines the imaging spectrums of IFs that are commonly encountered in LDCT. The prevalence, clinical significance, and recommended management in accordance with current guidelines will be discussed. This work aims to provide radiologists and clinicians with the knowledge necessary to optimize patient care in LDCT for LCS.

**Abstract:**

While low-dose computed tomography (LDCT) for lung cancer screening (LCS) has been recognized for its effectiveness in reducing lung cancer mortality, it often simultaneously leads to the detection of incidental findings (IFs) unrelated to the primary screening indication. These IFs present diagnostic and management challenges, potentially causing unnecessary anxiety and further invasive diagnostic procedures for patients. This review article provides an overview of IFs encountered in LDCT, emphasizing their clinical significance and recommended management strategies. We categorize IFs based on their anatomical locations (intrathoracic–intrapulmonary, intrathoracic–extrapulmonary, and extrathoracic) and discuss the most common findings. We highlight the importance of utilizing guidelines and standardized reporting systems by the American College of Radiology (ACR) to guide appropriate follow-ups. For each category, we present specific IF examples, their radiologic features, and the suggested management approach. This review aims to provide radiologists and clinicians with a comprehensive understanding of IFs in LCS for accurate assessment and management, ultimately enhancing patient care. Finally, we outline a few key aspects for future research and development in managing IFs.

## 1. Introduction

Lung cancer screening (LCS) with low-dose computed tomography (LDCT) has demonstrated the potential to reduce mortality in high-risk populations [1]. The recently updated guidelines significantly expand the eligibility criteria for LCS, potentially making an additional 5 million Americans eligible for LDCT scans annually [2]. This broader eligibility may lead to the detection of incidental findings (IFs) in more patients. Significant IFs most frequently encountered in the National Lung Screening Trial were emphysema (43.0%), coronary artery calcification (12.1%), and masses (7.4%) [3]. These IFs often trigger a cascade of downstream evaluations, including referrals to sub-specialists, additional imaging studies, and potentially invasive diagnostic procedures. These evaluations can, in turn, cause significant economic burdens on the healthcare system and potentially cause psychosocial distress for patients due to anxiety and prolonged diagnostic uncertainty.

LDCT routinely scans a region extending from the lower neck to the upper abdomen. IFs can thus be generally categorized by their anatomical locations as (1) intrathoracic –intrapulmonary, (2) intrathoracic–extrapulmonary, and (3) extrathoracic. The reported prevalence of IFs deemed significant or warranting further investigation varied from 4.4% to 40.7%, with a higher likelihood in older individuals [4]. The 2022 version of the American College of Radiology (ACR) Lung Imaging Reporting and Data System (Lung-RADS) provides a structured approach to mitigate these challenges [5]. By utilizing an ‘S’ modifier for clinically significant incidental findings not directly related to lung cancer, Lung-RADS promotes standardized reporting and facilitates communication between radiologists and clinicians.

This review aims to examine the spectrum of IFs encountered in LDCT for LCS. A thorough understanding of the prevalence, clinical significance, and recommended management strategies for IFs is essential for optimizing patient care. All patient images in this article were approved by the Institutional Review Board (the IRB file No. is 202400978B0) for retrospective review. The images are presented solely for case illustration and were de-identified to protect patient privacy.

## 2. General Rule for IFs in LDCT

The American College of Radiology (ACR)’s ‘Quick Reference Guide for Incidental Findings on Lung Cancer Screening CT Examinations’ provides a helpful overview for the initial evaluation and management of these findings [6]. A key principle of IF assessment is the comparison of current and prior imaging studies to assess its stability or progression. The interpreting radiologist plays a crucial role in documenting all significant IFs in the radiology report along with recommended follow-up measures in the ‘Impression’ Section of the report. 

## 3. Intrathoracic—Intrapulmonary

### 3.1. Common Findings

Mild or subsegmental atelectasis is a common intrapulmonary finding and generally does not require further workup. However, emphysema or bronchial wall thickening may warrant evaluation by a primary care provider (PCP) with potential referral to a pulmonologist for a further assessment of underlying lung disease.

**Figure 1:**
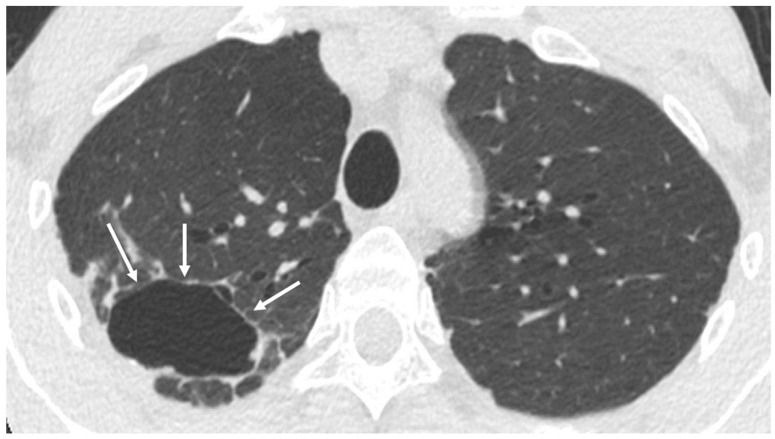
Incidental finding of right upper lobe (RUL) cavitary lesion in low-dose computed tomograph (LDCT). Axial LDCT of 41-year-old male demonstrates cavitary lesion measuring 5.4 cm in RUL (arrows). Further workup is recommended to determine etiology.

Pulmonary cysts can be attributed to aging, as these have been reported in up to 13% of individuals over 65 years of age [7]. Isolated, thin-walled (<2 mm) solitary cysts typically represent benign sequelae of a prior infection or inflammatory processes. However, thick-walled cysts (>2 mm) or those associated with a nodular component warrant further evaluation due to their potential association with cystic lung cancer (Figure 1).

### 3.2. Interstitial Lung Diseases (ILDs)

**Figure 2:**
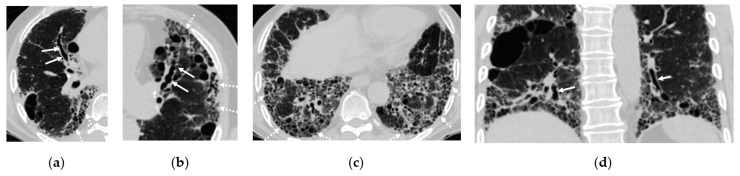
Interstitial lung disease found on a computed tomography (CT) scan of a 72-year-old male with hypertension, cough, and history of smoking. (**a**–**c**) Axial CT scans demonstrate traction bronchiectasis in right middle lobe (arrows in **a**) and left upper lobe (arrows in **b**) as well as reticulations and cystic changes at the periphery of both lungs (dashed arrows in **a**–**c**). (**d**) Coronal CT scan reveals traction bronchiectasis (arrows) and basilar predominance of the cystic change or honeycombing.

Abnormal opacities associated with ILDs (Figure 2) that are frequently detected in LDCT include ground-glass opacities (GGOs) and reticulations. Specific ILD subtypes manifesting diffuse GGOs include cellular nonspecific interstitial pneumonia (NSIP), hypersensitivity pneumonitis, desquamative interstitial pneumonia, cryptogenic organizing pneumonia, sarcoidosis, and subacute diffuse alveolar damage. Two key fibrotic ILDs, usual interstitial pneumonia (UIP) and fibrotic NSIP, can manifest with reticulations in LDCT. UIP characteristically demonstrates a peripheral and basilar predominance, often accompanied by traction bronchiectasis and honeycombing within fibrotic areas. These features are incorporated into the Fleischner Society’s diagnostic criteria, which categorizes UIP patterns as typical, probable, indeterminate, or alternative diagnoses [8]. Conversely, fibrotic NSIP, though also frequently basilar predominant, typically lacks honeycombing and may exhibit subpleural sparing, which is not typically observed in UIP. The identification of these findings in LDCT should prompt pulmonary subspecialist referral and a consideration of high-resolution computed tomography (HRCT) [9].

### 3.3. Bronchiectasis

**Figure 3:**
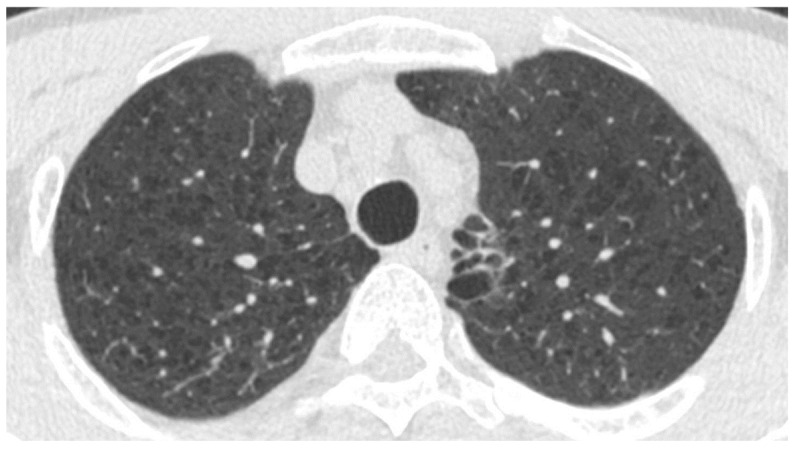
An axial low-dose computed tomograph of a 63-year-old male demonstrating centrilobular and paraseptal emphysema predominantly in the bilateral upper lobes of the lung.

**Figure 4:**
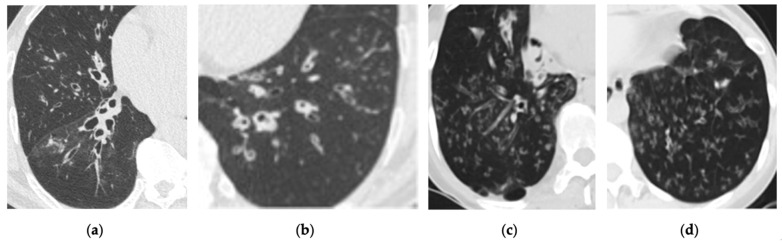
Incidental finding of bronchiectasis with centrilobular nodules associated with tree-in-bud pattern on low-dose computed tomography (LDCT) scan. (**a**,**b**) Axial LDCT scans of a 65-year-old male with chronic cough demonstrate bronchiectasis and findings of infectious bronchiolitis. (**c**,**d**) Axial LDCT scans of a 42-year-old female reveal similar findings in the bilateral lower lungs.

Bronchiectasis, characterized by the irreversible and progressive dilation of the airways due to chronic injury, is frequently observed in conjunction with emphysema in patients with chronic obstructive pulmonary disease [10] (Figure 3). The presence of this combination in a computed tomography (CT) image should warrant a PCP evaluation, as the extent of structural damage to lung parenchyma and small airways has been linked to an increased frequency of chronic obstructive pulmonary disease exacerbations [11]. The finding of bronchiectasis accompanied by peribronchial micronodules in LDCT may suggest infectious bronchiolitis (Figure 4). In such cases, the findings could be correlated with the patient’s clinical symptoms, and a PCP evaluation for the assessment of an underlying infection is warranted.

### 3.4. Cystic Lung Disease

Cystic lung disease refers to the presence of well-defined, thin-walled, air-filled spaces that displace centrilobular structures towards the periphery. The two most prevalent cystic lung diseases are lymphangioleiomyomatosis and pulmonary Langerhans cell histiocytosis. Lymphangioleiomyomatosis is a systemic neoplastic disease characterized by the proliferation of atypical smooth muscle cells. Lymphangioleiomyomatosis can be sporadic or associated with tuberous sclerosis complex. The findings in LDCT include large numerous thin-walled cysts, which can progressively increase in size and number in follow-up exams. In contrast, pulmonary Langerhans cell histiocytosis typically affects young adult males with a history of smoking. Characteristic LDCT findings include reticulonodular opacities and thin-walled cysts predominantly distributed in the mid and upper lung zones, with sparing of the costophrenic recesses and the costomediastinal recesses. The identification of either condition warrants a referral to a pulmonologist for further evaluation [9].

## 4. Intrathoracic–Extrapulmonary

### 4.1. Coronary Artery Calcifications (CAC)

**Figure 5:**
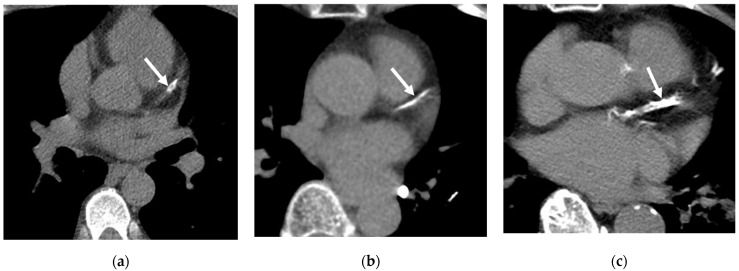
Qualitative assessment of coronary artery calcification (CAC) by low-dose computed tomography (LDCT). Axial LDCT scans demonstrate varying degrees of CAC. (**a**) Mild CAC with isolated flecks of calcification (arrow) in left anterior descending artery (LAD). (**b**) Moderate CAC with more extensive calcification (arrow) involving two segments of LAD. (**c**) Severe CAC with continuous, circumferential calcification (arrow) of LAD.

Coronary artery calcification (CAC) is a well-recognized predictor of cardiovascular events and can be readily detectable in LDCT [12]. Although the precise quantification of CAC scores may be challenging with LDCT, especially due to variations in the slice thickness and reconstruction kernel, a qualitative assessment can be a valuable tool for stratifying cardiovascular risk [13,14,15]. This can be achieved through either an overall visual assessment of the entire coronary arterial territories or a segmented vessel-specific approach, categorizing the CAC severity as none, mild, moderate, or severe (Figure 5). These qualitative methods have demonstrated comparable performance to Agatston scoring in association with the risk assessment of coronary heart disease and all-cause mortality [15]. Therefore, the presence of CAC in LDCT should prompt a referral to a PCP for a comprehensive assessment of the atherosclerotic cardiovascular disease risk.

### 4.2. Aorta and Pulmonary Artery

**Figure 6:**
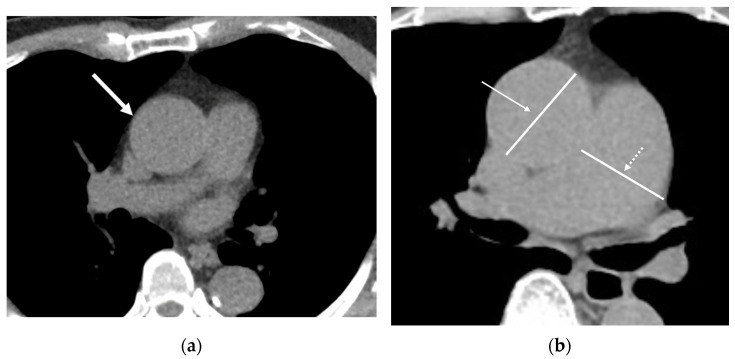
The incidental findings of abnormalities in the cardiovascular system on low-dose computed tomograph (LDCT). (**a**) An axial LDCT of a 64-year-old male with hypertension demonstrates a dilated ascending aorta (AA, arrow), which measures 48 mm in diameter. The size of the AA, though not exceeding the aneurysm threshold of 50 mm, warrants further assessment and a potential referral to a cardiologist due to its size exceeding 42 mm. (**b**) An axial LDCT of a 71-year-old female demonstrates an ascending aortic dilatation (arrow) measuring 45 mm and a dilated main pulmonary artery (MPA, dashed arrow) measuring 37 mm. MPA dilation, exceeding 31 mm, is potentially associated with pulmonary arterial hypertension and warrants further evaluation by a cardiologist or pulmonologist.

Although aortic diseases represent a relatively low proportion (3.4%) of IFs in LDCT, aortic aneurysms constitute the vast majority of these aortic findings [16]. The aortic diameter is influenced by gender, age, and body surface area. The upper normal limit for the ascending aorta (AA) can be calculated with the formula D (mm) = 31 + 0.16 × age, and that for the descending aorta (DA) with the formula D (mm) = 21 + 0.16 × age. An AA diameter of 42 mm or greater in CT images typically warrants further assessment, including consultation with the PCP for possible aneurysm surveillance and potential referral to a cardiologist. While an AA diameter exceeding 42 mm may be considered ectatic or dilated, the term ‘aneurysm’ is generally reserved for diameters that exceed 150% of the normal expected size. This translates to approximately 5.0 cm for the AA and 4.0 cm for the DA [17] (Figure 6).

Similarly, the diameter of the main pulmonary artery (PA) should be assessed in LDCT. Main PA dilation is a recognized indicator of increased pulmonary artery pressure [18]. A main PA measuring 31 mm or greater or equal in diameter to the ascending aorta in the CT scan is considered dilated [6] (Figure 6). Therefore, the identification of main PA dilatation in LDCT warrants further evaluation by a PCP, with potential referral to a cardiologist or pulmonologist for the assessment of possible pulmonary arterial hypertension.

### 4.3. Aortic Valve Calcification (AVC)

**Figure 7:**
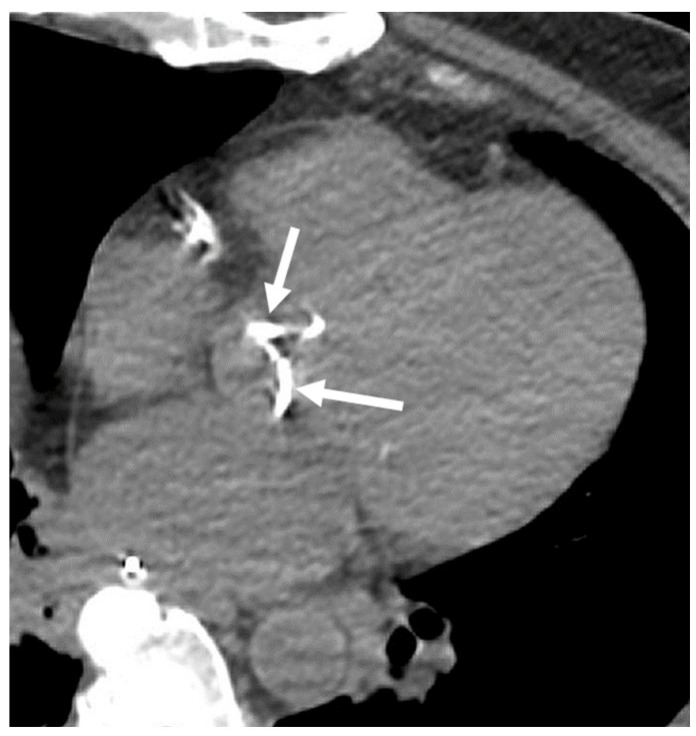
The incidental finding of aortic valve calcification on a computed tomograph (CT). An axial CT of a 65-year-old male demonstrates calcification of the aortic valve leaflets (arrows).

The presence and severity of AVC can predict the likelihood of developing heart failure and the potential need for valvular replacement. The prevalence of AVC increases with age, affecting 29% of individuals over 65, 40% of those over 75, and 48–57% of individuals over 80 [19,20]. Notably, the progression of AVC appears to be an independent predictor of coronary artery plaque progression, suggesting distinct mechanisms underlying these two processes [21]. However, a recent single-center study on coronary CT angiography found that the presence of AVC was significantly associated with a higher calcium score and higher coronary artery disease reporting and data system (CAD-RADS) 2.0 categories in the study [22] (Figure 7). Therefore, recognizing AVC in LDCT can identify individuals at higher risk for future cardiac events and thus prompt further evaluation by a PCP.

### 4.4. Pleura and Pericardium

**Figure 8:**
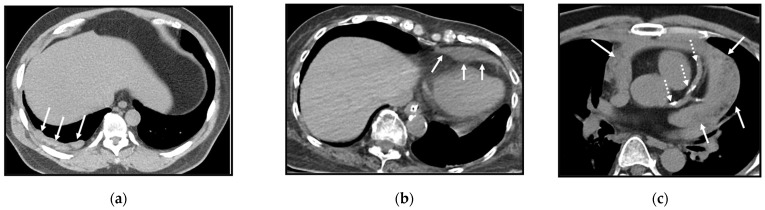
The incidental findings of the pleura and pericardium on a low-dose computed tomograph (LDCT). The LDCT shows (**a**) focal and irregular thickening of the right lower pleura (arrows) of a 46-year-old male; (**b**) asymmetrical thickening of the lower pericardium measuring 1.6 cm in thickness (arrows) of an 80-year-old female, where the normal pericardium thickness should be less than 4 mm; and (**c**) pericardial effusion measuring up to 3.1 cm in thickness (arrows) of a 39-year-old male. The incidental finding of left coronary artery calcification was also noted (dashed arrows).

Trace or small amounts of pericardial and pleural effusion are common findings in LDCT and typically require no further workup. Moderate or large pericardial and pleural effusions warrant clinical correlation to determine the underlying etiology (Figure 8). Potential explainable causes include autoimmune diseases, prior radiation therapy or infections, renal disease, and drugs [16].

The normal thickness of the pericardium in the CT scan is less than 4 mm, and a thickening of more than 4 mm may necessitate further investigation [16] (Figure 8).

Pericardial cysts are also occasionally encountered. Simple fluid-filled cysts with Hounsfield units (HU) ≤ 10 generally require no follow-up. However, higher attenuation cysts, especially those associated with symptoms like chest pain, may warrant further evaluation with MRI to assess for potential hemorrhage, infection, or the compression of adjacent structures [16].

### 4.5. Thymus

Incidental anterior mediastinal masses are estimated to occur in 0.4% of high-risk smokers over the age of 40 [23]. The classic differential diagnosis for anterior mediastinal masses includes thyroid mass, thymic hyperplasia, thymoma, teratomas, or malignant tumors such as thymic carcinoma or lymphoma.

**Figure 9:**
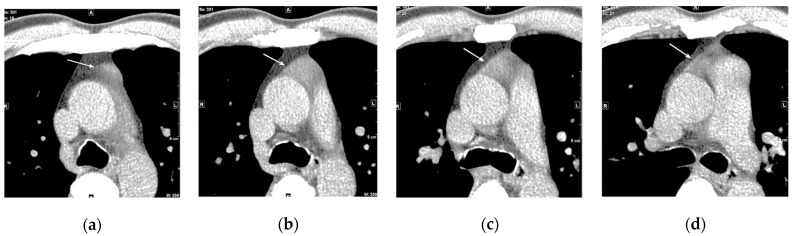
The incidental finding of soft tissue in the anterior mediastinum in a 53-year-old male. (**a**–**d**) Sequential axial low-dose computed tomographs demonstrating a homogeneous soft tissue density (arrows) in the prevascular space, raising the differential diagnoses of thymoma, thymic hyperplasia, or thymic remnant. It was interpreted as a thymic remnant. (A, anterior. P, posterior).

In a CT scan, a normal thymic remnant (Figure 9) can sometimes be observed, particularly in women aged 40–69, as they exhibit less fatty replacement of the thymus compared to men in this age group [24]. Factors such as cigarette smoking and a high body mass index are also associated with accelerated fatty replacement of the thymus [24]. In younger patients, a soft tissue mass conforming to the shape of the thymic gland often suggests thymic hyperplasia, particularly if there is a history of chemotherapy, radiation therapy, or corticosteroid use. Diagnostic uncertainty may be resolved with a chemical shift (opposed-phase) MRI or follow-up CT in three months [16]. In individuals over the age of 40, thymoma should be considered for an anterior mediastinal mass, especially if accompanied by myasthenia gravis. Conversely, a large, heterogeneous mass exhibiting local invasion, lymphadenopathy, and pleural effusion is concerning for aggressive thymic epithelial neoplasms, such as thymic carcinoma or carcinoid. While lymphoma can also present with local lymphadenopathy, it can be differentiated from other mediastinal masses by a characteristic infiltrative growth pattern and encasing but not invading vascular structures.

### 4.6. Mediastinal Lymph Nodes

**Figure 10:**
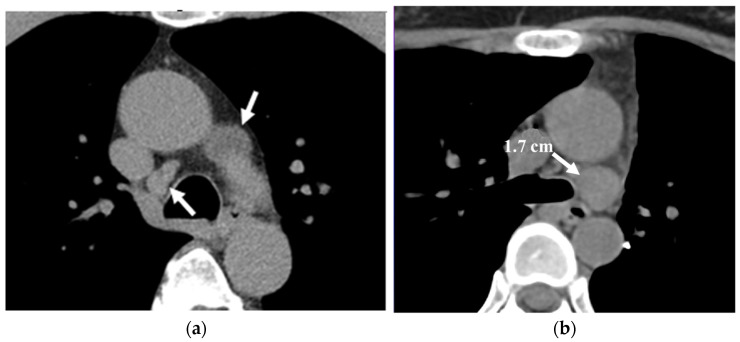
Mediastinal lymph nodes (LNs) in a low-dose computed tomography (LDCT) scan for lung cancer screening. (**a**) An axial LDCT image of a 61-year-old male shows nodes, with each measuring less than 15 mm in short-axis diameter (arrows), typically requiring no further workup. (**b**) An axial LDCT image of a 40-year-old female showing a node measuring 1.7 cm in short-axis diameter (arrow), warranting surveillance or a follow-up contrast-enhanced chest CT within 3–6 months in the absence of an identifiable cause.

Incidental mediastinal lymphadenopathy is a relatively common finding in LCS, with a reported prevalence of 1% to 3% in LDCT [25,26]. Mediastinal lymph nodes measuring 15 mm or greater in a short-axis diameter warrant further evaluation by a PCP (Figure 10). An assessment should consider underlying conditions that could explain the lymphadenopathy, such as emphysema, interstitial lung disease, sarcoidosis, or cardiac disease. If no readily identifiable cause is found, a broader differential diagnosis should be explored, including lymphoma, undiagnosed metastatic disease, and infection. A follow-up contrast-enhanced chest CT within 3–6 months may be considered to assess for changes and guide further management [16].

### 4.7. Esophagus

**Figure 11:**
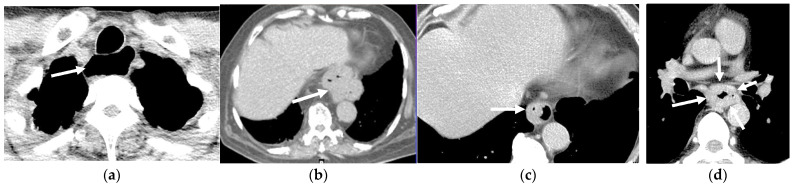
The incidental esophageal findings in low-dose computed tomography (LDCT) scans. (**a**) The dilated esophagus at the upper third of the thoracic level (arrow) of a 61-year-old male. (**b**) A conspicuous hiatal hernia in the lower mediastinum (arrow) of a 79-year-old female. (**c**) Asymmetrical thickening of the distal esophageal wall (arrow) of a 67-year-old male. (**d**) Circumferential esophageal wall thickening at the middle third of the thoracic level of a 52-year-old male, raising suspicion of esophageal cancer (arrows).

The incidental findings related to the esophagus are not uncommon in LDCT. These findings can include esophageal dilation, achalasia, or the presence of a large hiatal hernia (Figure 11). While these IFs are often benign, they may warrant further evaluation by a PCP. Furthermore, focal esophageal wall thickening or a mass (Figure 11), even being suspected on a non-contrast CT, should prompt a referral to a gastroenterologist for additional diagnostic workup, as these may indicate significant pathology including esophageal cancer. In a cohort of 24 patients with esophageal abnormalities reported in LDCT, 18 underwent an endoscopic evaluation, revealing two newly diagnosed esophageal adenocarcinomas [27].

## 5. Extrathoracic

### 5.1. Neck (Thyroid)

Chest CT accounts for the majority of incidental thyroid nodule detections of all diagnostic modalities, as the scan often includes the lower neck region [28]. Furthermore, the prevalence of thyroid nodules increases with age, aligning with the demographic group commonly targeted for LCS with LDCT.

Nodular hyperplasia is the most common benign etiology, accounting for over 90% of thyroid nodules. However, approximately 5% of nodules harbor malignancy, with papillary and follicular carcinomas being the most prevalent types. The risk factors for thyroid carcinoma include an age of less than 20 or greater than 60 years, a history of neck irradiation, and a family history of thyroid cancer. The prognosis of thyroid cancer varies widely depending on the cell type. Papillary and follicular carcinomas generally have a favorable outcome, with 5-year survival rates of more than 99.5% for both. Medullary thyroid carcinoma is more aggressive, with 5-year survival ranging from 43% to 99.5% depending on the stage at diagnosis. Anaplastic thyroid carcinoma is the most aggressive form, carrying a dismal 5-year survival rate of only 8% [29].

**Figure 12:**
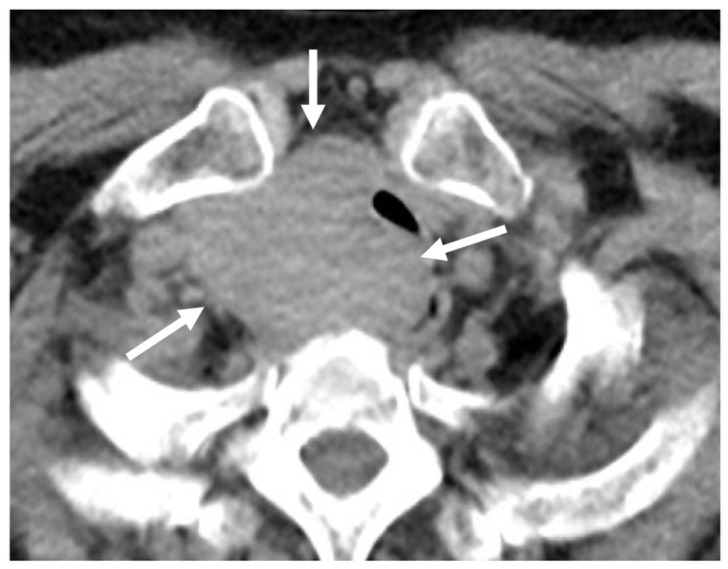
Incidental thyroid mass in low-dose computed tomograph (LDCT). Axial non-contrast LDCT of 66-year-old female demonstrates large, homogeneous right thyroid mass (arrows) with mass effect and marked narrowing of tracheal lumen.

In an LDCT scan, large and heterogeneous thyroid lesions likely represent goiter (Figure 12) and may not require immediate intervention, but thyroid function testing may be considered. Similarly, diffuse infiltrative thyroid lesions are predominantly benign with the exception of lymphoma, leukemia, and rare cases of metastasis. Nodules smaller than 15 mm are typically benign and require no further workup. However, distinct nodules 15 mm or larger warrant further evaluation with thyroid ultrasound (US) and a clinical assessment [30].

The ACR published the Thyroid Imaging, Reporting and Data System (TI-RADS) to standardize the assessment and reporting of thyroid nodules in US [31]. Several sonographic features can raise suspicion for malignancy in thyroid nodules detected in US. These include hypoechogenicity, microcalcifications, central vascularity, lobulated or irregular margins, extension beyond the thyroid border, an incomplete halo sign, a taller-than-wide shape (which suggests an anteroposterior diameter larger than the transverse diameter), and documented nodule enlargement over time.

Ectopic thyroid tissue, while typically found at the base of the tongue, can occur anywhere along the thyroglossal duct tract, extending down to the mediastinum, even in the presence of a normally located thyroid gland [32]. In a CT scan, ectopic thyroid tissue in the mediastinum typically appears as a hyperdense, enhancing soft tissue mass, similar to the normal thyroid gland [33]. However, lymphoma, thymic tumors, and germ cell tumors should be considered in differential diagnoses. If ectopic thyroid tissue is suspected, a functional assessment with radionuclide imaging using technetium-99m pertechnetate, iodine-123, or iodine-131 can confirm the presence of radioiodine uptake by the thyroid tissue [33]. Ectopic parathyroid glands can similarly be found in various locations, including the retropharyngeal space, thyroid gland, mediastinum, and within the carotid sheath near the angle of the mandible [34].

### 5.2. Breast

**Figure 13:**
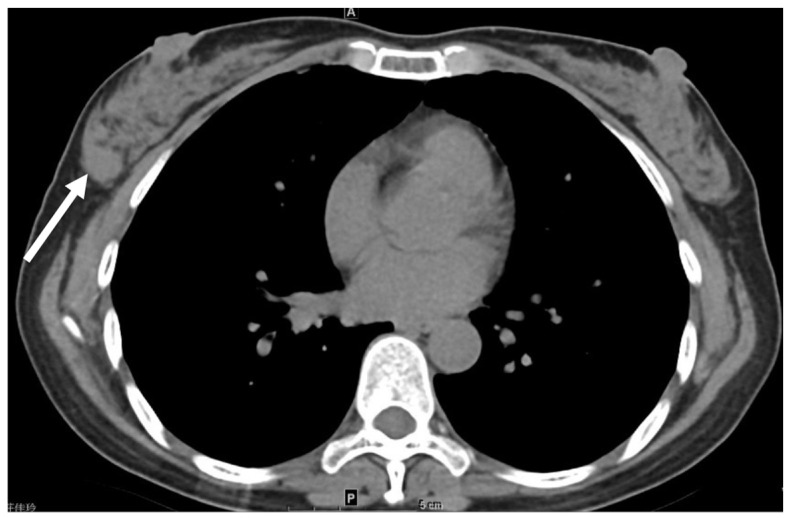
An incidental solid breast nodule detected in a low-dose computed tomograph (LDCT). An axial LDCT of a 48-year-old female shows a 1.5 cm solid nodule in the right outer breast (arrow). The nodule was proven to be breast cancer after surgery and pathology. (A, anterior. P, posterior).

**Figure 14:**
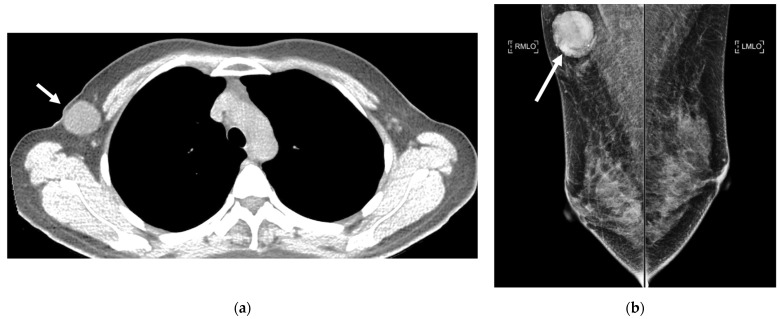
An incidental breast mass in a low-dose computed tomograph (LDCT). (**a**) An axial LDCT of a 48-year-old female demonstrates a 3.3 cm right breast mass (arrow). (**b**) A mammogram revealing a corresponding mass at the right upper breast (arrow), which was subsequently confirmed as a benign phyllodes tumor by surgery and pathology. (RMLO, right mediolateral oblique view. LMLO, left mediolateral oblique view).

Although CT is not the primary imaging modality for breast evaluation, it can sometimes be the first to reveal a new primary breast cancer. The reported prevalence of breast cancer among incidental lesions detected in CT ranged from 24% to as high as 70% [35]. In LDCT, coarse calcifications and simple cysts without a solid component are typically considered benign and require no further workup. However, any other type of nodule, mass, or asymmetric density warrants further evaluation with diagnostic mammography or US (Figure 13 and Figure 14). Additionally, malignant lesions were also reported to be significantly larger than benign lesions in a large series (mean of 28.5 mm vs. 20.2 mm, *p* < 0.05) [35].

### 5.3. Upper Abdomen

Given that LDCT scans typically include the upper abdomen to fully visualize the bilateral lower lungs, incidental lesions can also be detected in solid organs in the upper abdomen including the liver, pancreas, spleen, adrenal glands, kidneys, and gastrointestinal tract.

#### 5.3.1. Liver

**Figure 15:**
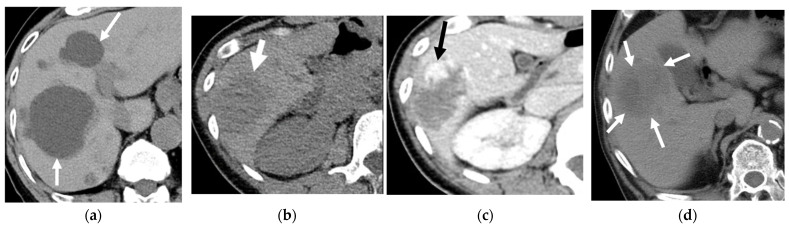
Incidental liver lesions in low-dose computed tomography (LDCT) scans. (**a**) Simple hepatic cysts (arrows). (**b**) A LDCT scan of a 40-year-old female showing a 4.9 cm hypodense mass in the right lobe liver (arrow). (**c**) A dynamic contrast-enhanced CT of the liver was arranged. The CT scan reveals peripheral nodular and centripetal enhancement, suggesting hemangioma (arrow). (**d**) A 5.3 cm hypodense mass (arrows) in the LDCT scan of a 56-year-old male was ultimately proven to be hepatocellular carcinoma.

**Figure 16:**
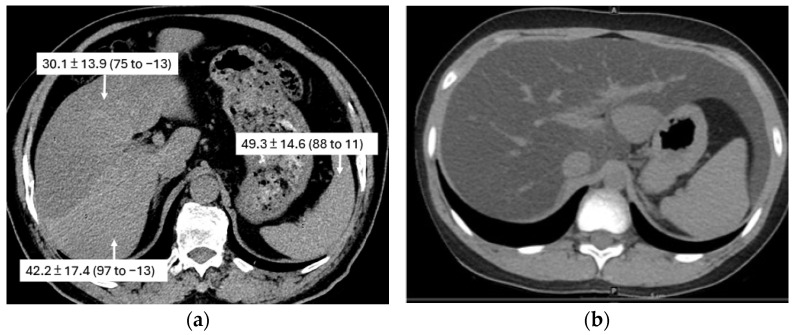
The incidental finding of fatty liver in low-dose computed tomography (LDCT) scans. On the non-enhanced CT scan, the normal liver density is 8 to 10 HU higher than that of the spleen. (**a**) An axial LDCT scan of a 52-year-old male illustrates segmental fatty liver infiltration predominantly in the right lobe. The attenuation values, provided in Hounsfield units (HU), are shown in the scan across different regions. These measurements highlight that the fatty liver in the anterior aspect (30.1 ± 13.9 HU) is much more conspicuous than that in the posterior aspect (42.2 ± 17.4 HU). The attenuation value of the spleen is 49.3 ± 14.6 HU. (**b**) An axial LDCT scan of a 39-year-old female reveals diffuse and marked fatty changes in the entire liver.

**Figure 17:**
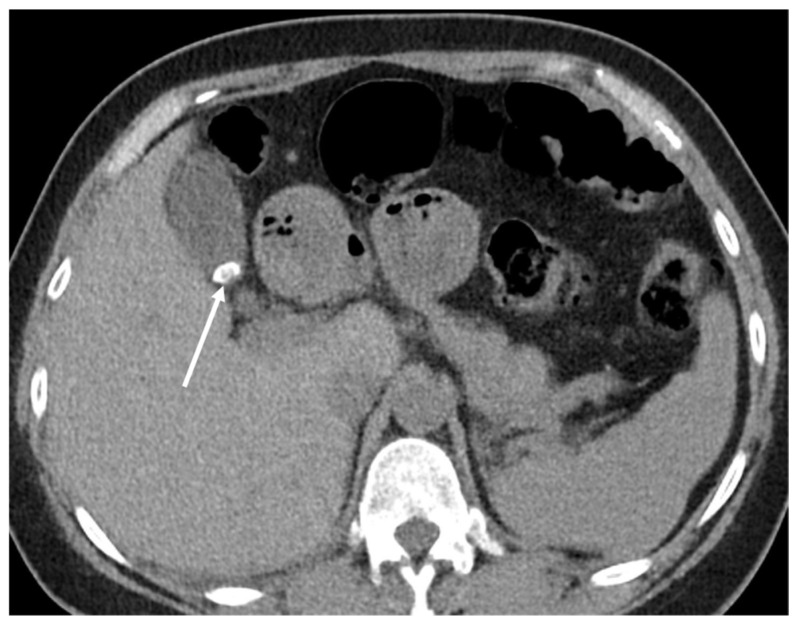
An axial low-dose computed tomography scan of a 52-year-old female demonstrating an incidental gallstone (arrow).

The most frequently encountered benign hepatic lesions in LDCT scans are cysts, hemangiomas, and focal nodular hyperplasia (Figure 15). These typically appear as focal hypodense lesions [36]. A study reported a 34% prevalence of hepatic cysts in patients undergoing LDCT [37]. Simple cysts smaller than 1 cm are considered benign and require no further workup. However, soft tissue nodules or masses measuring 1 cm or greater warrant further investigation with contrast-enhanced abdominal CT or MRI to assess for potential malignancy (Figure 15). Diffuse hepatic lesions could sometimes be observed. These include fatty liver (hepatic steatosis), which appears brighter than the spleen (the normal liver density is 8 to 10 HU higher than that of the spleen), or liver cirrhosis (Figure 16). These findings should prompt an evaluation by a PCP for underlying disease and appropriate management. These patients may also present with incidental gallstones, which should be documented in the LDCT report to alert both the PCP and the patient as it may precipitate an acute episode of cholecystitis due to the obstruction of the cystic duct (Figure 17).

#### 5.3.2. Adrenal Gland

**Figure 18:**
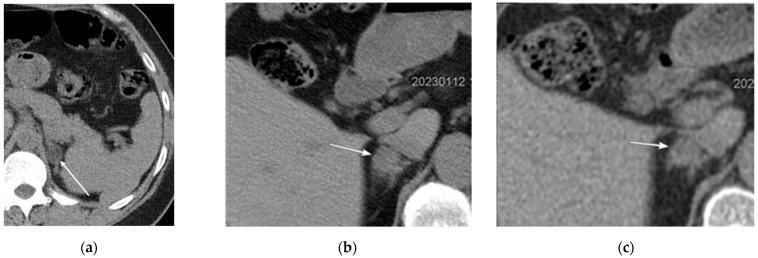
Incidental findings of adrenal nodules on an axial low-dose computed tomography (LDCT) scan. (**a**) An axial LDCT scan of a 52-year-old female demonstrating a left adrenal adenoma with fat density measuring 17 × 11 mm (arrow). (**b**,**c**) Axial LDCT scans of a 46-year-old male demonstrating a stable right adrenal nodule measuring 1.8 cm (arrow) with no interval change observed in the follow-up CT 14 months later (shown in (**c**)).

Incidental adrenal lesions detected in LDCT can be evaluated based on the following criteria: calcification and fat-containing nodules (<10 HU), which could represent adenoma, are generally considered benign and require no further workup (Figure 18). Similarly, soft tissue nodules smaller than 1 cm or adrenal nodules that have remained stable for at least one year in prior imaging are typically not concerning (Figure 18). However, any other adrenal nodule or mass with atypical features or indeterminate characteristics warrants further investigation with contrast-enhanced adrenal CT or MRI to assess for potential malignancy [38].

#### 5.3.3. Kidney

**Figure 19:**
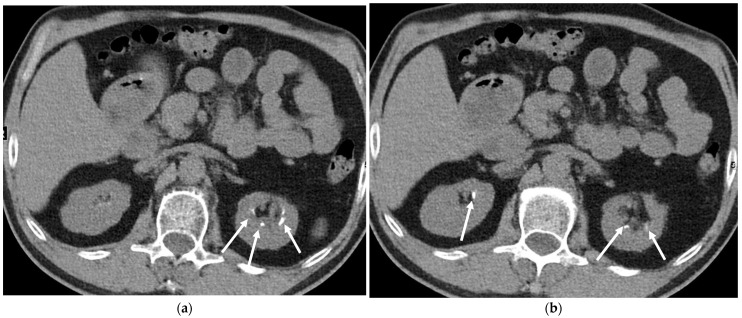
(**a**,**b**) Axial low-dose computed tomography scans of a 63-year-old male demonstrating bilateral renal calculi (arrows).

**Figure 20:**
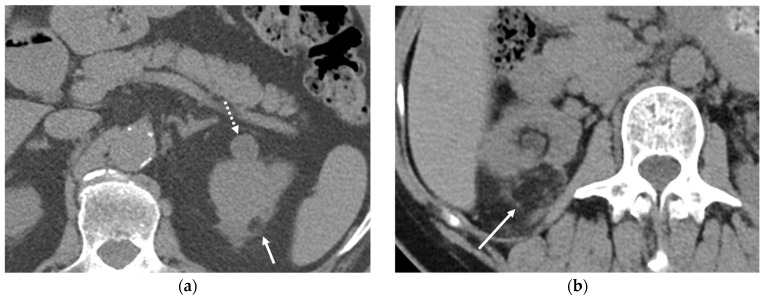
Incidental renal findings in low-dose computed tomography (LDCT) scans. (**a**) An axial LDCT scan of a 67-year-old male demonstrating a 9 mm fat-containing nodule (arrow) in the left kidney, suggesting angiomyolipoma. But the incidental finding of the soft tissue nodule (dashed arrow) warrants further evaluation. (**b**) An axial LDCT scan of a 49-year-old female showing an exophytic right upper renal angiomyolipoma (arrow) measuring 4.6 cm in diameter, which may be associated with a risk of spontaneous rupture.

Incidental renal lesions are a common finding in LDCT (Figure 19). Non-obstructing renal calculi require no further workup. However, soft tissue or mixed-density renal masses, regardless of size, warrant further investigation with contrast-enhanced CT or MRI to assess for malignancy (Figure 20).

The management of incidental renal masses detected on non-contrast CT, without visible fat, follows a decision tree based on the recommendation of the ACR [39]: (1) Homogeneous masses that are too small to characterize are likely benign cysts and require no further workup. (2) Homogeneous masses with a thin or imperceptible wall can be assessed for their density. Masses with −10 to 20 HU or with 70 or greater HU are likely benign and hemorrhagic cysts, respectively. These cysts are also categorized as Bosniak I or II. (3) Homogeneous masses with 21 to 69 HU or heterogeneous masses with a thick or irregular wall, mural nodules, septa, or calcification are indeterminate and necessitate further evaluation with contrast-enhanced CT or MRI.

In addition to the conditions above, the presence of renal lesions with specific characteristics, such as angiomyolipoma (Figure 20), may also warrant further imaging due to its risk of spontaneous rupture.

#### 5.3.4. Pancreas

Common IFs in the pancreas, such as coarse calcifications, are typically benign and do not require further imaging. However, the presence of a cyst or mass, regardless of size or morphology, warrants an additional evaluation with contrast-enhanced abdominal CT or MRI to assess for potential malignancy or other underlying pathology [6].

### 5.4. Bone

**Figure 21:**
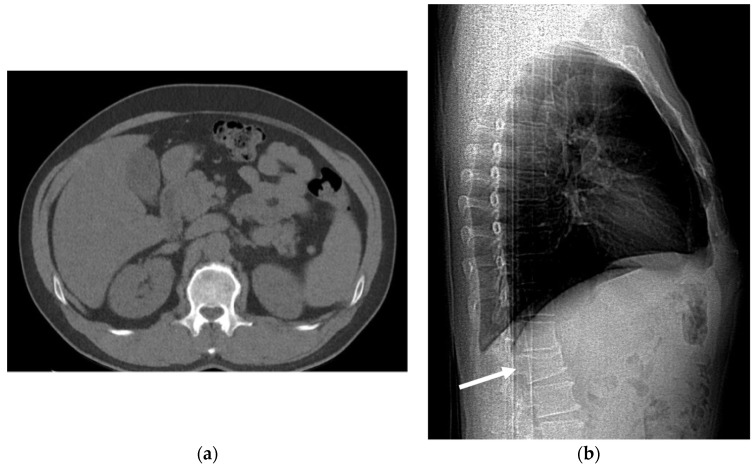
(**a**) An axial low-dose computed tomography (LDCT) scan of a 56-year-old male at the level of the first lumbar vertebra demonstrating no significant musculoskeletal abnormalities. (**b**) A lateral scout view of the same LDCT scan clearly revealing the compression fracture of the first lumbar vertebral body (arrow), highlighting the importance of a comprehensive scan review for accurate diagnosis.

While primarily intended for lung cancer screening, LDCT has also demonstrated utility in assessing bone conditions that can be related to all-cause mortality in LCS populations. For instance, a randomized controlled trial demonstrated a 51% prevalence of vertebral fractures among LCS participants who died during a median follow-up of 6 years compared to a 35% prevalence in survivors [40]. Additionally, each 10 HU decline in the trabecular bone density of the vertebrae was independently associated with an 8% increased risk of mortality. Another study has shown that the CT attenuation values of the trabecular bone in thoracic vertebrae can be used to predict low bone mineral density, a significant risk factor for osteoporosis and fractures [41]. Specifically, CT value thresholds have been established for identifying individuals at risk, with a greater predictive value being observed in women, older individuals, and those with lower heights and weights. These findings suggest that LDCT could potentially serve as an opportunistic screening tool for osteoporosis in LCS populations, enabling early identification and intervention. Therefore, it is crucial to incorporate bone assessment into LDCT interpretation. While degenerative changes are common, a vertebral bone density measurement of less than 100 HU at the first lumbar vertebra indicates osteoporosis and warrants a PCP evaluation as well as the consideration of a dual-energy X-ray absorptiometry scan [6]. Bone densities between 100 and 130 HU, suggesting osteopenia, may prompt an evaluation for preventive measures and potential treatment options. Scout view or coronal CT reconstructions can facilitate the detection of subtle compression fractures that may be difficult to visualize on axial CT images alone (Figure 21). Importantly, incidental sclerotic or lytic bone lesions identified in LDCT in a patient with cancer history should involve the assessment of potential metastasis. Bone islands, another common incidental finding, can be differentiated from untreated osteoblastic metastases using CT attenuation values. A cutoff of 881 HU has been reported to distinguish between these two entities, with values below this threshold suggesting metastasis [42].

## 6. Summary of Critical Values and Significant Findings Needing Further Surveillance

Nodules or lesions under 1 cm in the liver and adrenal glands, thyroid nodules under 1.5 cm, and mediastinal lymph nodes with a short axis diameter under 1.5 cm are often considered benign and may not necessitate further workup. Similarly, non-obstructing renal calculi and simple or hemorrhagic renal cysts (Bosniak I or II) under 4 cm are typically not concerning.

A dilated aorta (aorta > 4.2 cm), dilated pulmonary trunk (>3.1 cm), calcification of the aortic valve or coronary arteries, solid breast nodules, esophageal lesions, pleural or pericardial thickening or effusion, and fatty liver are among the findings that necessitate further evaluation and surveillance.

## 7. Future Directions

As LCS expands to include a broader range of age groups and risk factors, the detection and management of IFs will continue to be crucial aspects of clinical practice and research. While the ACR has published guidelines for managing IFs, several key aspects may still require future endeavors in both clinical practice and research:

1. A standardized terminology and reporting system for IFs would significantly enhance communication between radiologists and clinicians, or even between institutions for patients seeking a second opinion. This can ensure accurate risk assessment and consistent management strategies.
2. Developing risk stratification models that incorporate both IF characteristics and individual risk factors, such as age, smoking history, and cardiovascular comorbidities, could lead to more personalized management strategies.
3. Artificial intelligence (AI)-based tools hold the promise of improving workflow efficiency and enhancing detection and risk stratification for LDCT [43]. This could involve developing AI algorithms that utilize computer-aided detection (CAD) systems to improve detection sensitivity and/or utilize radiomic features for the accurate characterization of IFs. Such advancements could streamline the interpretation process, improve diagnostic accuracy, and ultimately lead to better patient management.
4. Longitudinal studies could be conducted to evaluate the long-term impact of IFs on patient outcomes, including the risks of overdiagnosis and overtreatment. Understanding these long-term effects will be crucial for refining LCS guidelines and optimizing the balance of benefits and harms for individual patients.

## 8. Conclusions

In conclusion, while many IFs are benign and do not require further intervention, a subset of IFs may indicate clinically significant underlying pathology, necessitating further evaluation and management. A thorough understanding of the prevalence, clinical significance, and recommended management strategies for various IFs is crucial to effectively navigate through LDCT interpretation and optimize patient care.
