# Correction: Lin et al. Incidental Findings in Lung Cancer Screening. *Cancers* 2024, *16*, 2600

**DOI:** 10.3390/cancers17010041

**Published:** 2024-12-26

**Authors:** Yenpo Lin, Khulan Khurelsukh, I-Gung Li, Chen-Te Wu, Yi-Ming Wu, Gigin Lin, Cheng-Hong Toh, Yung-Liang Wan

**Affiliations:** 1 Department of Medical Imaging and Intervention, Chang Gung Memorial Hospital at Linkou, Taoyuan City 333, Taiwan; yenpojack@cgmh.org.tw (Y.L.); melik@cgmh.org.tw (C.-T.W.); m7075@cgmh.org.tw (Y.-M.W.); giginlin@cgmh.org.tw (G.L.); eldomtoh@cgmh.org.tw (C.-H.T.); 2 Department of Medical Imaging and Radiological Sciences, College of Medicine, Chang Gung University, Taoyuan City 333, Taiwan; h_ashka@yahoo.com; 3 Department of Medical Imaging and Intervention, New Taipei Municipal Tucheng Hospital, New Taipei City 236, Taiwan; ladenli@cgmh.org.tw

## Corrected Affiliation

The affiliation for K.K. has been updated to the Department of Medical Imaging and Radiological Sciences, College of Medicine, Chang Gung University, Taoyuan City 333, Taiwan. Additionally, affiliations 2 and 3 have been switched to ensure all authors’ affiliations are in correct order.

## Error in Figure and Figure Legend

In the original publication [1], errors were identified in Figures 2 and 4 as published.

- Figure 2: The original figure intended to illustrate interstitial lung disease (ILD) but inadequately highlighted bronchiectasis in Figure 2a. Additionally, Figure 2b,c presented confusing representations with the consolidation in the right lower lobe. The revised figure now accurately showcases the full spectrum of ILD findings, including traction bronchiectasis, reticulations, cystic changes, and honeycombing. Furthermore, the citation for Figure 2 has been corrected to ensure relevance to the surrounding text.
- Figure 4: The replacement figure provides a clearer and more focused depiction of bronchiectasis and infectious bronchiolitis.

The corrected Figure 2 and Figure 4, along with their updated corresponding figure legends, are provided below.

**Figure 2:**
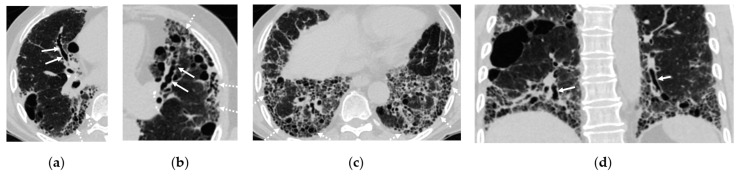
Interstitial lung disease found on a computed tomography (CT) scan of a 72-year-old male with hypertension, cough, and history of smoking. (**a**–**c**) Axial CT scans demonstrate traction bronchiectasis in right middle lobe (arrows in **a**) and left upper lobe (arrows in **b**) as well as reticulations and cystic changes at the periphery of both lungs (dashed arrows in **a**–**c**). (**d**) Coronal CT scan reveals traction bronchiectasis (arrows) and basilar predominance of the cystic change or honeycombing.

**Figure 4:**
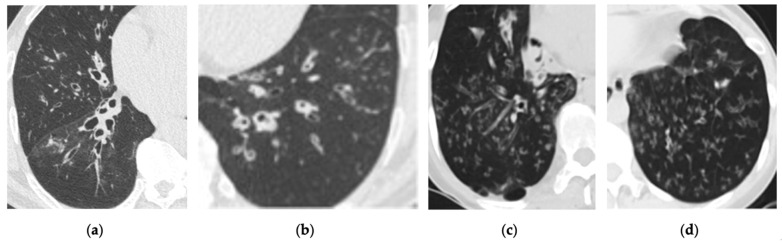
Incidental finding of bronchiectasis with centrilobular nodules associated with tree-in-bud pattern on low-dose computed tomography (LDCT) scan. (**a**,**b**) Axial LDCT scans of a 65-year-old male with chronic cough demonstrate bronchiectasis and findings of infectious bronchiolitis. (**c**,**d**) Axial LDCT scans of a 42-year-old female reveal similar findings in the bilateral lower lungs.

## Text Correction

There was an error in the original publication [1]. Figures 2 and 11 citations were missing from two paragraphs.

A correction has been made to Section 3. Intrathoracic—Intrapulmonary, Section 3.2. Interstitial Lung Diseases (ILDs), Paragraph Number 1, and Section 4. Intrathoracic–Extrapulmonary, Section 4.7. Esophagus, and Paragraph Number 1. The correct paragraphs are shown below.

Abnormal opacities associated with ILDs (Figure 2) that are frequently detected in LDCT include ground-glass opacities (GGOs) and reticulations. Specific ILD subtypes manifesting diffuse GGOs include cellular nonspecific interstitial pneumonia (NSIP), hypersensitivity pneumonitis, desquamative interstitial pneumonia, cryptogenic organizing pneumonia, sarcoidosis, and subacute diffuse alveolar damage. Two key fibrotic ILDs, usual interstitial pneumonia (UIP) and fibrotic NSIP, can manifest with reticulations in LDCT. UIP characteristically demonstrates a peripheral and basilar predominance, often accompanied by traction bronchiectasis and honeycombing within fibrotic areas. These features are incorporated into the Fleischner Society’s diagnostic criteria, which categorizes UIP patterns as typical, probable, indeterminate, or alternative diagnoses [8]. Conversely, fibrotic NSIP, though also frequently basilar predominant, typically lacks honeycombing and may exhibit subpleural sparing, which is not typically observed in UIP. The identification of these findings in LDCT should prompt pulmonary subspecialist referral and a consideration of high-resolution computed tomography (HRCT) [9].

The incidental findings related to the esophagus are not uncommon in LDCT. These findings can include esophageal dilation, achalasia, or the presence of a large hiatal hernia (Figure 11). While these IFs are often benign, they may warrant further evaluation by a PCP. Furthermore, focal esophageal wall thickening or a mass (Figure 11), even being suspected on a non-contrast CT, should prompt a referral to a gastroenterologist for additional diagnostic workup, as these may indicate significant pathology including esophageal cancer. In a cohort of 24 patients with esophageal abnormalities reported in LDCT, 18 underwent an endoscopic evaluation, revealing two newly diagnosed esophageal adenocarcinomas [27].

The authors apologize for any inconvenience caused and reiterate that the scientific conclusions are unaffected. This correction was approved by the Academic Editor. The original publication has also been updated.
